# Adipose-derived mesenchymal stem cells differentiate into heterogeneous cancer-associated fibroblasts in a stroma-rich xenograft model

**DOI:** 10.1038/s41598-021-84058-3

**Published:** 2021-02-25

**Authors:** Yoshihiro Miyazaki, Tatsuya Oda, Yuki Inagaki, Hiroko Kushige, Yutaka Saito, Nobuhito Mori, Yuzo Takayama, Yutaro Kumagai, Toutai Mitsuyama, Yasuyuki S. Kida

**Affiliations:** 1 Department of Gastrointestinal and Hepato-Biliary-Pancreatic Surgery, Faculty of Medicine, University of Tsukuba, 1-1-1 Tennodai, Tsukuba, Ibaraki 305-8575 Japan; 2 Cellular and Molecular Biotechnology Research Institute, National Institute of Advanced Industrial Science and Technology (AIST), Central 5-41, Higashi 1-1-1, Tsukuba, Ibaraki 305-8565 Japan; 3 Artificial Intelligence Research Center, National Institute of Advanced Industrial Science and Technology (AIST), 2-4-7 Aomi, Koto-ku, Tokyo, 135-0064 Japan; 4 AIST-Waseda University Computational Bio Big-Data Open Innovation Laboratory (CBBD-OIL), 3-4-1 Okubo, Shinjuku-ku, Tokyo, 169-8555 Japan; 5 Graduate School of Frontier Sciences, University of Tokyo, 5-1-5 Kashiwanoha, Kashiwa, Chiba 277-8561 Japan; 6 Advanced Photonics and Biosensing Open Innovation Laboratory, National Institute of Advanced Industrial Science and Technology (AIST), Central 5-41, Higashi 1-1-1, Tsukuba, Ibaraki 305-8565 Japan

**Keywords:** Mesenchymal stem cells, Cancer microenvironment, Cancer models, Gastrointestinal cancer, Cancer microenvironment, Cancer models, Gastrointestinal cancer, Pancreatic disease, Pancreatic cancer

## Abstract

Cancer-associated fibroblasts (CAFs) are the key components of the densely proliferated stroma in pancreatic ductal adenocarcinoma (PDAC) and contribute to tumor progression and drug resistance. CAFs comprise heterogeneous subpopulations playing unique and vital roles. However, the commonly used mouse models have not been able to fully reproduce the histological and functional characteristics of clinical human CAF. Here, we generated a human cell-derived stroma-rich CDX (Sr-CDX) model, to reproduce the clinical tumor microenvironment. By co-transplanting human adipose-derived mesenchymal stem cells (AD-MSCs) and a human PDAC cell line (Capan-1) into mice, the Sr-CDX model recapitulated the characteristics of clinical pancreatic cancer, such as accelerated tumor growth, abundant stromal proliferation, chemoresistance, and dense stroma formed from the heterogeneous CAFs. Global RNA sequencing, single-cell based RNA sequencing, and histological analysis of CAFs in the Sr-CDX model revealed that the CAFs of the Sr-CDX mice were derived from the transplanted AD-MSCs and composed of heterogeneous subpopulations of CAF, including known and unknown subtypes. These lines of evidences suggest that our new tumor-bearing mouse model has the potential to address an open question in CAF research, that is the mechanism of CAF differentiation.

## Introduction

Pancreatic ductal adenocarcinoma (PDAC) has one of the worst outcomes among all cancers, with a 5-year survival rate less than 10%^1^. In general, PDAC is asymptomatic during its early stages, which makes early diagnosis quite difficult. In many cases, patients are often diagnosed late during the disease progression, which limits therapeutic options and usually forces clinicians to initiate multi-drug systemic chemotherapy regimens. However, most patients receive only temporary benefits from this approach and are prone to acquire chemotherapy resistance^2,3^.

The tumor microenvironment (TME) has been reported to play a major role in chemotherapy resistance in PDAC^4^. The TME is composed of the extracellular matrix (ECM), cancer-associated fibroblasts (CAFs), immune cells, microvessels, etc., of which CAFs are a major component^5,6^. CAFs facilitate tumor growth via release of soluble signaling cytokines, and attenuation of drug responses and immunosurveillance by producing various ECM proteins^7,8^. From pioneering studies on the presence of molecular and functional CAF heterogeneity in PDAC^9,10^, it is known that the heterogeneity of CAFs is regulated by signals derived from cancer cells in distinct microenvironmental conditions. Indeed, CAFs are composed of diverse subpopulations such as: (1) myoblastic CAFs (myCAFs), which are located immediately adjacent to the cancer cells and demonstrate elevated αSMA expression; (2) inflammatory CAFs (iCAFs), which are located further away from the cancer cells and characterized by the secretion of inflammatory mediators, such as Interleukin-6 (IL-6), but with low α-smooth muscle actin (αSMA) expression^11^; and (3) antigen-presenting CAFs (apCAFs), which express major histocompatibility complex (MHC) class II and CD74, allowing antigen-dependent T-cell receptor ligation in CD4+ T cells^10^. However, the mechanisms underlying the differentiation of CAFs remain unclear^12^.

Since PDAC in genetically engineered mouse models (GEMMs) is not the same as the human PDAC, a convenient in vivo model that reproduces TME and human CAFs is essential for research on CAFs. However, the commonly used mouse models, including cell line-derived xenografts (CDXs) and patient-derived xenografts (PDXs), are unable to simulate stromal proliferation and histological characteristics in clinical PDAC^13,14^. Stroma in CDXs is usually scant, and cancer cells proliferate in a medullary pattern; this is distinct from the pattern observed in clinical PDAC, i.e., a glandular formation pattern. PDXs display non-uniform characteristics depending on the features of the original patient-derived cancer tissues; therefore, standardized experiments cannot be applied. Hence, a new in vivo model is urgently needed for investigating CAFs that contribute to the poor prognosis of notorious PDAC.

Various origins of CAFs in PDAC are proposed, including adipose-derived mesenchymal stem cells (AD-MSCs)^15–17^, bone marrow-derived mesenchymal stem cells (BM-MSCs)^18–21^, pancreatic stellate cells (PSCs)^22^, and cancer cells themselves by epithelial–mesenchymal transition^23–25^. Although multiple origins of CAFs have been reported, it is unclear whether CAFs in clinical pancreatic cancer are of a single origin or multiple origins. AD-MSCs are known to possess a high degree of plasticity in their differentiation potential^26^. In addition, we have previously established in vitro experimental systems to differentiate AD-MSCs into myCAFs and iCAFs by co-culturing with pancreatic cancer cells^27^. Therefore, we focused on AD-MSCs as the source of CAFs, which would allow differentiation into CAFs in vivo.

In this study, we generated CDX mice with AD-MSCs and investigated whether AD-MSCs, among the various candidates for CAF precursors, could differentiate into multiple heterogeneous CAF subpopulations such as myCAFs, iCAFs, and apCAFs. We found that the Sr-CDX model using AD-MSCs accurately reproduced the pathological features, chemotherapy resistance, and CAF heterogeneity observed in the clinical PDAC.

## Results

### Co-transplantation of adipose-derived MSCs and human PDAC cell line induced high tumor growth and chemotherapy resistance

**Figure 1:**
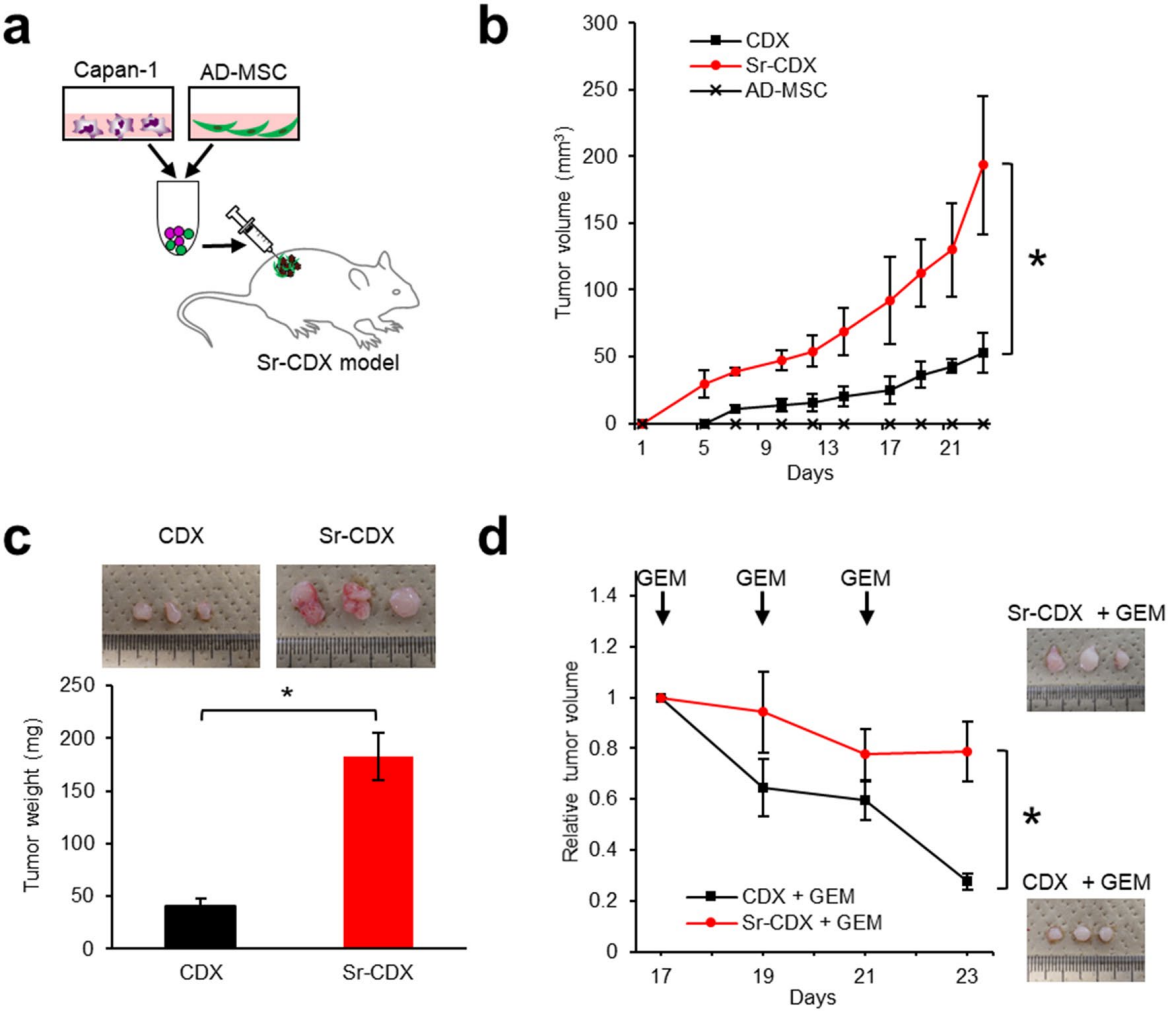
Co-transplantation of adipose-derived MSCs (AD-MSCs) with a human PDAC cell line, inducing accelerated tumor growth and increased chemotherapy resistance. (**a**) Schematic illustration of the Sr-CDX model. A human PDAC cell line (Capan-1) and AD-MSCs were mixed and co-transplanted subcutaneously into the flank of immunodeficient mice. (**b**) The tumor growth curve and (**c**) excised tumor weights at day 23. Results show the mean ± SD (n = 3). *P < 0.05, one-way analysis of variance (ANOVA) with Tukey's method. (**d**) The tumor growth curve with gemcitabine treatment and images of the harvested tumors at day 23. Results show the mean ± SD (n = 3). *P < 0.05, unpaired Student’s t-test. GEM, gemcitabine.

To obtain an in vivo model that mimics clinical PDAC characteristics (i.e., stromal-rich and capable of cancer gland formation)^13,28^, a human PDAC cell line (Capan-1) was co-transplanted with several commercially available human MSCs (Fig. 1a). Among adipose-, bone marrow-, or umbilical cord tissue-derived human MSCs, AD-MSCs exhibited the most prominent tumor-promoting effect and histology similar to the clinical PDAC (Fig. S1)^27^. AD-MSC was therefore selected as a source of human stromal cells to develop a new mouse model. Tumors in the Sr-CDX mice were significantly larger than those in the conventional CDX (Capan-1 only) mice throughout the observation period (Fig. 1b). Notably, the average tumor weight in the Sr-CDX group was approximately four times greater than that in the conventional CDX group at day 23 (Fig. 1c). AD-MSCs alone did not induce tumor formation. Similar results were obtained using another cell line, MIAPaCa-2 (Fig. S2a,b). The chemotherapy resistance of Sr-CDX mice, a burden of clinical PDAC, which is largely attributed to the role of the stromal desmoplasia, was then assessed. The Sr-CDX and conventional CDX (Capan-1 only) groups were treated thrice with gemcitabine through intraperitoneal administration. The effect of gemcitabine was clearly limited in the Sr-CDX group as opposed to the CDX group, recapitulating the chemotherapy resistance in Sr-CDX mice (Fig. 1d).

### Co-transplantation of human adipose-derived MSCs and Capan-1 cells recapitulated the dense stroma and cancer gland formation, histological characteristics of human PDAC

**Figure 2:**
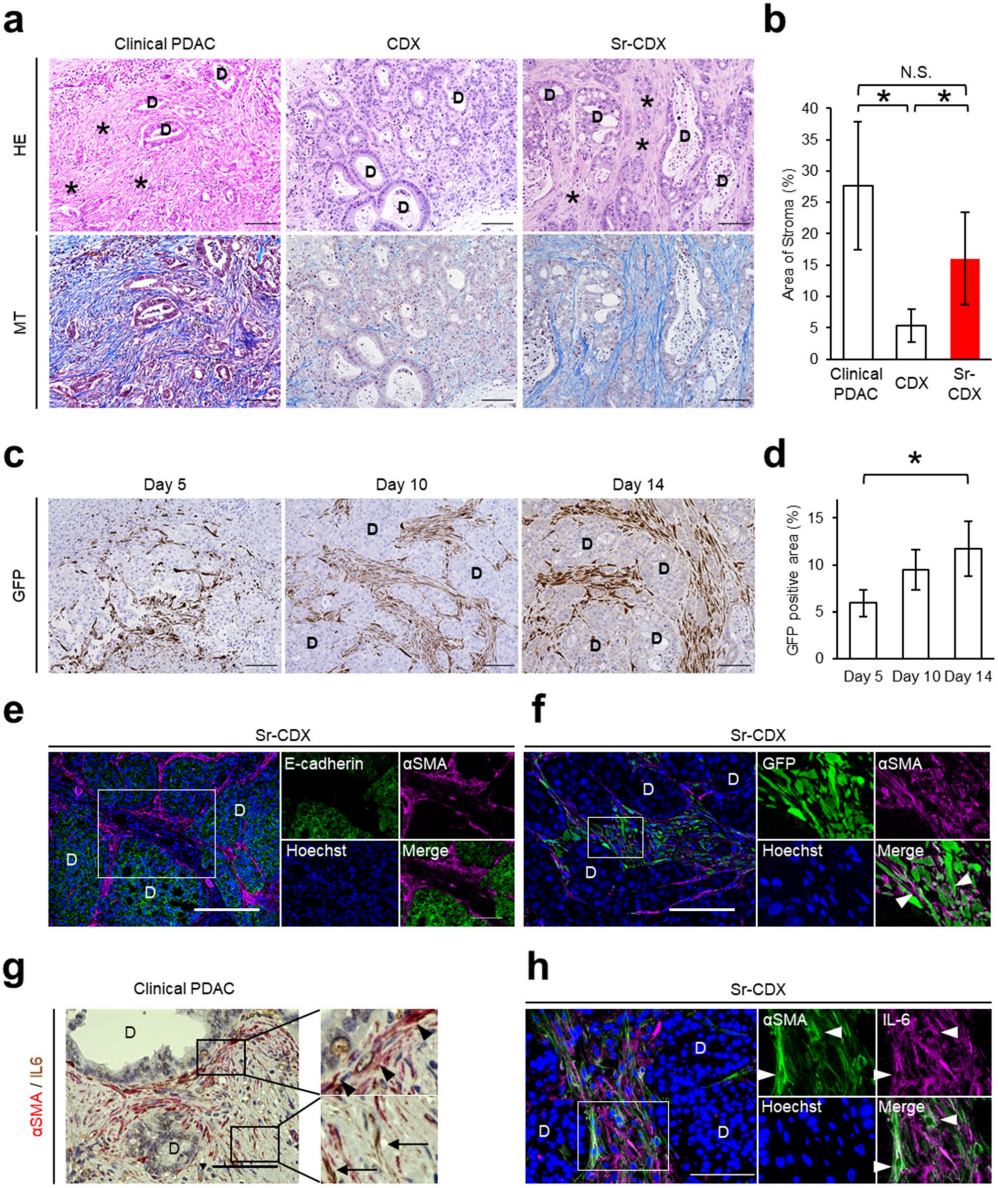
Co-transplantation of adipose-derived human MSCs recapitulated the histological morphology of human PDAC. (**a**) Representative images of H&E staining and Masson’s trichrome staining in clinical PDACs, the CDX model, and the Sr-CDX model. D, cancer duct formation. *, stromal area. Scale bars, 100 µm. (**b**) Quantification of the stromal area in clinical PDACs, CDX, and Sr-CDX groups. Results show the mean ± SD (n = 5). *P < 0.05, one-way ANOVA with Tukey's method. N.S., not significant. (**c**) Representative IHC image of GFP in Sr-CDX tumors at days 5, 10, and 14. Hematoxylin (blue) was used as a nuclear counterstain. D, cancer duct structure. Scale bars, 100 µm. (**d**) Quantification of the GFP-positive area in each group (n = 5). Results show the mean ± SD *P < 0.05, one-way ANOVA with Tukey's method. (**e** and **f**) Representative IF image of Sr-CDX tumors. Left, a zoomed-out image. The small plots on the right show a magnification of the depicted area within the selection (white rectangle). D, cancer duct structure. (**e**) Each plot is stained for E-cadherin (green), αSMA (magenta), and Hoechst 33,342 (blue). Scale bar, 100 µm. (**f**) Each plot is stained for αSMA (magenta), GFP (green), and Hoechst 33,342 (blue). Arrowheads indicate both αSMA- and GFP-positive cells. Scale bar, 50 µm. (**g**) Representative IHC image of human PDAC stained for αSMA (red) or IL-6 (brown). Hematoxylin (blue) was used as a nuclear counterstain. Left, a zoomed-out image. The small plots on the right show a magnification of the area depicted in the selection (black rectangle). Arrowheads indicate αSMA-positive CAFs. Arrows indicate IL-6-positive CAFs. D, cancer duct structure. Scale bar, 100 µm. (**h**) Representative IF image of Sr-CDX tumors. Left, a zoomed-out image. The small plots on the right show a magnification of the area depicted in the selection (white rectangle). Each plot is stained for αSMA (green), IL-6 (magenta), and Hoechst 33,342 (blue). Arrowheads indicate both αSMA- and IL-6-positive CAFs. D, cancer duct structure. Scale bar, 100 µm.

In contrast to the conventional CDX mice, in which the stromal amount is quite poor, Sr-CDX mice showed morphological similarity with human clinical PDAC tumors, which involves an abundant stroma surrounding the ductal glands consisting of cancer cells (Fig. 2a). When the stromal fibrosis was highlighted using blue color with Masson’s trichrome (MT) staining (Fig. 2a) and quantified, the stromal collagen area in Sr-CDX mice was significantly larger than that in the CDX mice (Fig. 2b). Co-transplantation of MIAPaCa-2 with AD-MSCs showed an increase in the stromal area, but the formation of glandular tubular structures was not seen (Fig. S2c).

It is interesting to note whether the origin of stromal cells in Sr-CDX is the transplanted AD-MSCs or other cells of the mouse host. To confirm the transplanted AD-MSCs, the cells were labeled with green fluorescent protein (GFP) in lentiviral transduction^27^. The evolution of stromal cells over time could be traced by immunohistochemistry (IHC) against GFP. It was confirmed that almost all stromal cells in the Sr-CDX mice were GFP-positive, indicating that the dense stromal cells originated from the transplanted AD-MSCs and its derivatives (Fig. 2c). The GFP-positive stromal area under the total visual field gradually increased until day 14 (Fig. 2d). It was also explored whether the AD-MSCs differentiated into CAFs in Sr-CDX mice. IHC of the CAF marker αSMA revealed that αSMA-positive cells were located in close proximity to the E-cadherin-positive cancer cells, indicating that these are possibly myCAFs (Fig. 2e), and that these αSMA-positive myCAFs were derived from the GFP-positive AD-MSCs (Fig. 2f). CAFs in human clinical PDAC are known to be heterogeneous, including the αSMA-positive myCAFs located adjacent to the ductal glands and IL-6-positive iCAFs located apart from the cancer cells (Fig. 2g). It was observed that stromal cells in the Sr-CDX mice exhibited a immunohistochemical profile similar to that of the human PDACs, such as αSMA-positive, IL-6 positive, or double-positive profiles (Fig. 2h), indicating that the stromal cells in Sr-CDX mice are the CAFs that differentiated from the AD-MSCs (Sr-CDX CAFs).

### Gene-expression patterns of Sr-CDX CAFs showed known CAF-related gene profiles

**Figure 3:**
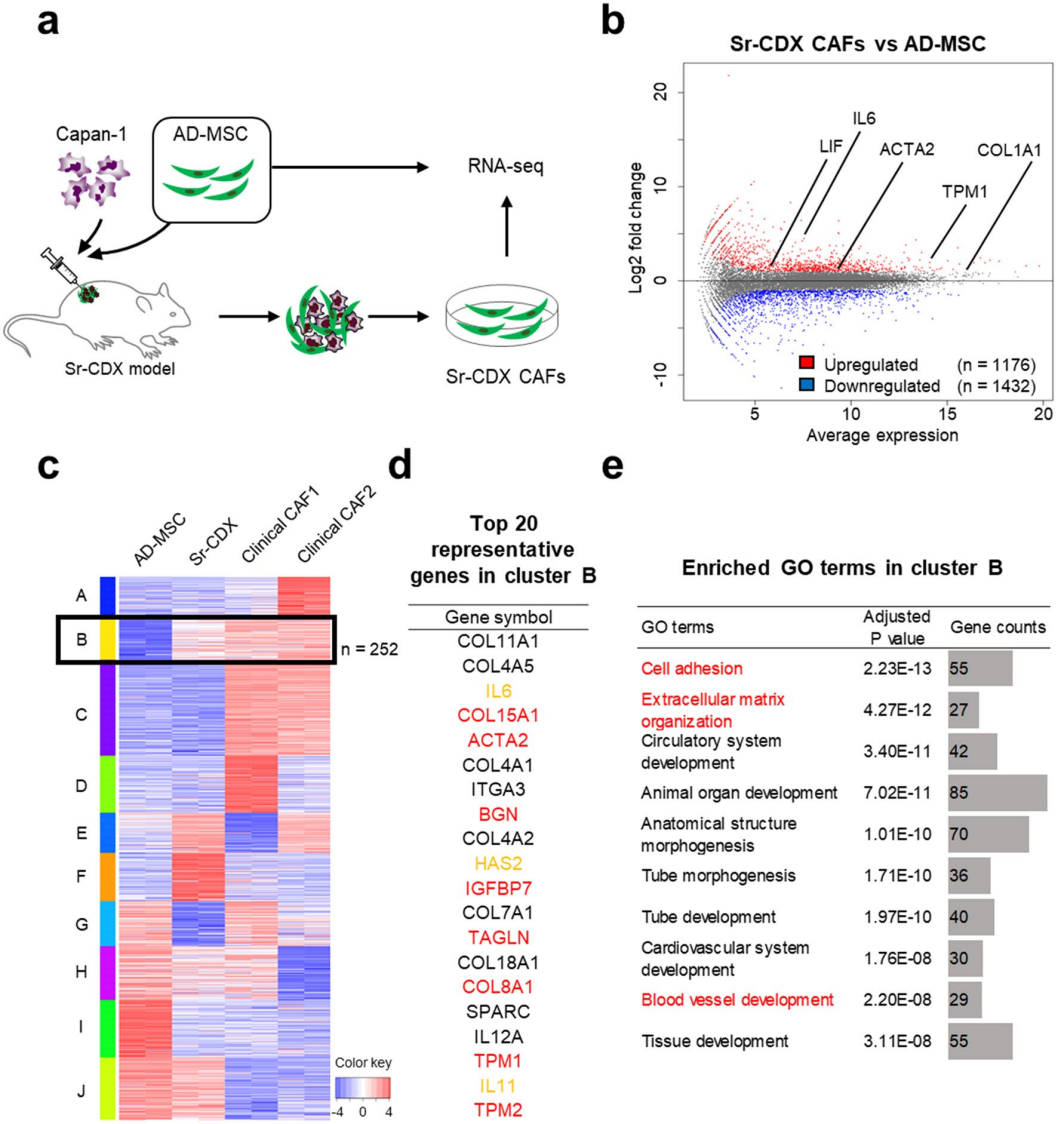
RNA-seq analysis revealed CAF-like differentiation of AD-MSCs in Sr-CDX mice. (**a**) Schematic illustration of the Sr-CDX model and the experimental design. The transcript profiles of Sr-CDX CAFs and original mono-cultured AD-MSCs were compared. Sr-CDX CAFs were obtained using the outgrowth method. (**b**) MA plot, a scatter plot of log twofold-change versus the average expression, showing differentially expressed genes (adjusted *P* < 0.05 and log2 [fold-change] ≥ 2) in Sr-CDX CAFs compared to the corresponding gene expression in the original AD-MSCs. Upregulated genes are shown in red (n = 1176), and downregulated genes are shown in blue (n = 1432). Representative upregulated CAF markers are indicated in the figure. (**c**) RNA-seq analysis of the original AD-MSCs (n = 2), Sr-CDX CAFs (n = 2), and clinical CAFs (n = 2). Heatmap of k-means clustering shows genes in two conditions: (1) upregulated in Sr-CDX CAFs compared to the expression in the original AD-MSCs and (2) strongly expressed in the two clinical CAFs and regarded as CAF-like gene expression in Sr-CDX. Cluster B, which matched these conditions and therefore should be regarded as a CAF-related gene signature, included 252 genes (black box). (**d**) List of the 20 most upregulated genes in Sr-CDX CAFs compared to the corresponding gene expression in the original AD-MSCs in cluster B. Red and yellow characters indicate myCAF and iCAF marker genes, respectively. (**e**) GO terms (biological process) enriched in cluster B are shown. Red characters indicate GO terms with possible association with CAF function, such as ECM remodeling or vascularization.

RNA sequencing (RNA-seq) analysis was performed to investigate the global gene-expression patterns of the original mono-cultured AD-MSCs, Sr-CDX CAFs, and clinical CAFs. The latter two were obtained using the outgrowth method (Fig. 3a). When transcriptomes of the Sr-CDX CAFs and original AD-MSCs were compared, 2608 genes were differentially expressed, of which 1176 were upregulated and 1432 downregulated in Sr-CDX (Fig. 3b). These upregulated genes included the iCAF and myCAF markers such as *IL-6, Leukemia Inhibitory Factor (LIF), actin alpha 2 (ACTA2),* and *Tropomyosin (TPM1)* (Fig. 3b, Fig. S3, Table S1).

To narrow down the list of CAF-related gene signatures in the Sr-CDX CAFs from 1176 genes, clustering analysis was performed by adding two clinical CAFs (Fig. 3c). After selecting the clusters involving the genes upregulated in Sr-CDX CAFs, compared to those of the original AD-MSC (cluster B, E, and F in Fig. 3c), the expression pattern was clearly observed in both clinical CAFs. The B cluster contained 252 genes (black box in Fig. 3c, Table S2). These 252 genes included the myCAF markers such as *ACTA2* and *COL15A1* (Fig. 3d in red characters) and iCAF markers such as *IL-6* and *hyaluronan synthase 2 (HAS2,* Fig. 3d in green characters, Table S2)^10^. To investigate the functional changes, Gene Ontology (GO) enrichment analysis was performed with the 252 genes in cluster B. The representative GO terms identified in the biological process category were “cell adhesion” (*p* = 2.23 E-13), “extracellular matrix organization” (*p* = 4.27 E-12), “blood vessel development” (*p* = 2.20 E-8), and “blood vessel morphogenesis” (*p* = 1.09 E-7, Fig. 3e and Table S3).

### Single-cell RNA-seq (scRNA-seq) analysis of Sr-CDX CAFs showed AD-MSCs differentiated into CAF subpopulations

**Figure 4:**
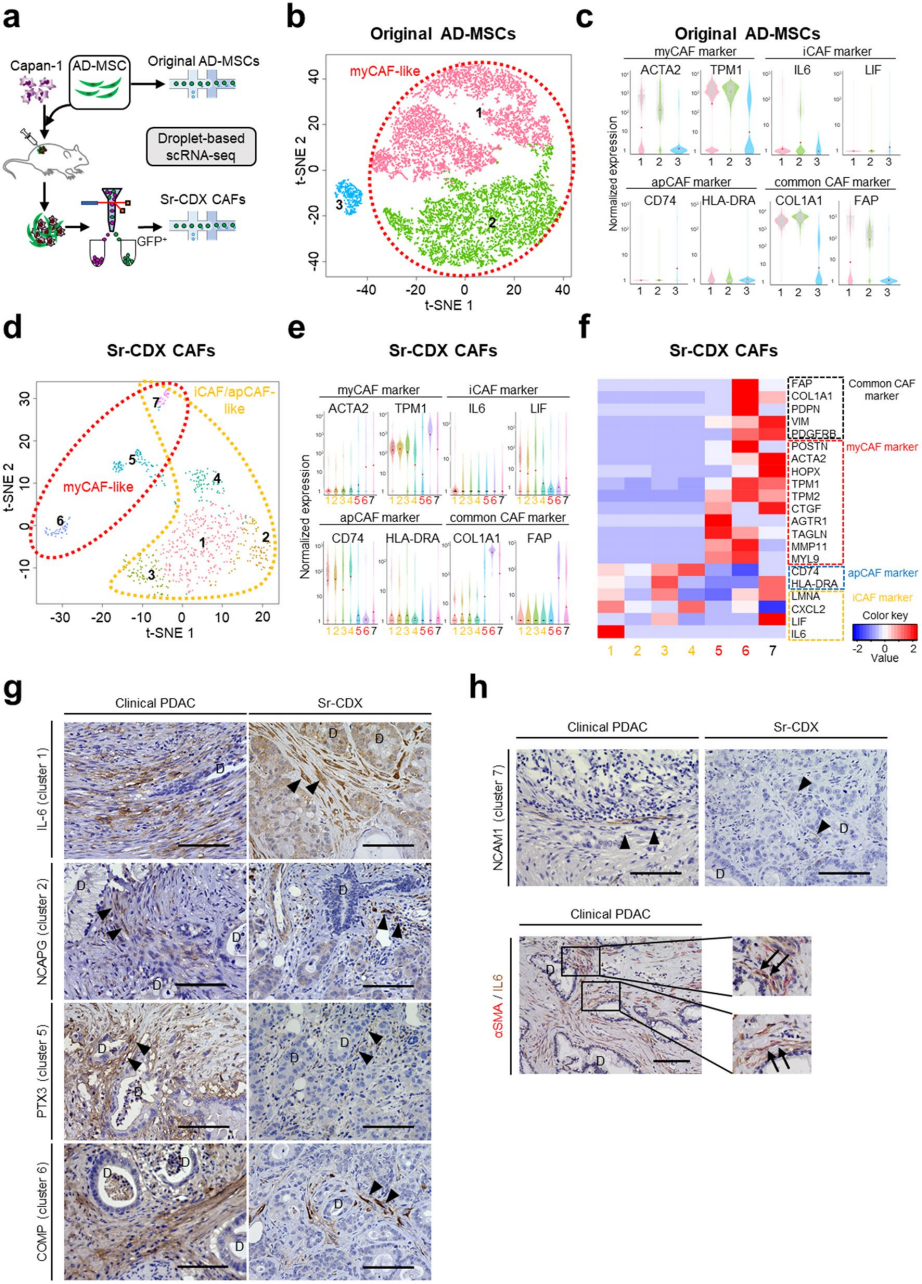
Single-cell RNA-seq (scRNA-seq) of Sr-CDX CAFs revealed the differentiation of AD-MSCs into CAF subpopulations. (**a**) Schematic illustration of the scRNA-seq analysis platform. The transcript profiles of Sr-CDX CAFs were compared with those of the original AD-MSCs. Sr-CDX CAFs labeled with GFP were obtained by tumor digestion, followed by FACS, and viable CAFs were analyzed with droplet-based scRNA-seq. (**b** and **c**) Original AD-MSCs; (**b**) clustering of represented t-distributed stochastic neighbor embedding (t-SNE) plot formed three clusters. (**c**) Violin plot of marker genes for myCAFs (*ACTA2* and *TPM1*), iCAFs (*IL-6* and *LIF*), apCAFs (*CD74* and *HLA-DRA*), and common CAFs (*COL1A1* and *FAP*). Red plots indicate median expression level. The myCAF marker expression was observed mainly in clusters 1 and 2. Clusters 1 and 2 were defined as “myCAF-like cluster.” (**d**–**f**) Sr-CDX CAFs; (**d**) clustering of t-SNE plots formed seven clusters. (**e**) Violin plot of various marker genes for myCAFs, iCAFs, apCAFs, and common CAFs. Red plots indicate median expression level. (**f**) Heatmap showing different expression levels of CAF markers among the seven clusters. The iCAF and apCAF markers were expressed mainly in clusters 1–4 and 7, whereas myCAF markers were expressed mainly in clusters 5–7. iCAF, apCAF, myCAF, and common CAF marker expression was identified in cluster 7. Clusters 1–4 were defined as iCAF/apCAF-like clusters, clusters 5 and 6 as myCAF-like clusters, and cluster 7 as myCAF/iCAF/apCAF-like cluster. (**g** and **h**) Representative IHC image of human PDAC and Sr-CDX tumors. (**g**) Each plot is stained for cluster markers IL-6 (cluster 1), NCAPG (cluster 2), PTX3 (cluster 5), and COMP (cluster 6). (**h**) Each plot is stained for cluster 7 marker NCAM1 (upper image) and stained for αSMA (red) and IL-6 (brown, lower image). The small plots on the right show a magnification of the indicated area (black rectangle). Hematoxylin (blue) was used as a nuclear counterstain. Arrowheads indicate each cluster marker positive CAF. Arrows indicate αSMA/IL-6 double-positive CAFs. D, cancer duct structure. Scale bar, 100 µm.

Sr-CDX CAFs were examined at the single-cell level by performing scRNA-seq. Briefly, soon after harvesting and digesting the tumors comprising GFP-labeled AD-MSCs and red fluorescent protein (RFP)-labeled Capan-1 cells, fluorescence-activated cell sorting (FACS) was used to isolate viable GFP-positive Sr-CDX CAF cells (Fig. 4a). The gene signatures of original mono-cultured AD-MSCs, including 10,994 single cells, revealed that AD-MSCs formed three large clusters with unique gene signatures, according to the t-distributed stochastic neighbor embedding (t-SNE) analysis (Fig. 4b). The myCAF marker genes (*ACTA2* and *TPM1*) and common CAF marker genes (*COL1A1* and *fibroblast activation protein alpha, FAP*) were highly expressed in clusters 1 and 2, whereas expression of iCAF- or apCAF-related marker genes was not observed in the original AD-MSCs (Fig. 4b,c, and Fig. S4a,b). Therefore, clusters 1 and 2 were defined as “myCAF-like clusters” (Fig. 4b).

The gene signatures of Sr-CDX CAFs containing 699 single GFP-positive cells formed seven distinct clusters according to t-SNE analysis, showing increased subpopulations (Fig. 4d). The common CAF marker genes (*COL1A1 and FAP*) and myCAF marker genes (*ACTA2* and *TPM1*) were highly expressed in clusters 5–7, whereas iCAF marker genes (*IL-6* and *LIF*) and apCAF marker genes (*CD74* and *HLA-DRA*) were highly expressed in clusters 1–4 and 7 (Fig. 4e and Fig. S4c). The gene-expression levels of several reported CAF markers in seven clusters were investigated^9–11^; the heat map showed varying levels of CAF marker expression among the seven clusters (Fig. 4f). In clusters 1–4, iCAF and apCAF markers were expressed at different levels, whereas myCAF and common CAF markers were not expressed. In cluster 5, myCAF markers were highly expressed, whereas other CAF marker expression levels were relatively low. In cluster 6, both myCAF and common CAF marker expression levels were high. In cluster 7, myCAF marker was highly expressed; iCAF (*LIF* and *LMNA*), apCAF (HLA-DRA), and common CAF markers were also expressed. Clusters 1–4 were defined as iCAF/apCAF-like clusters, clusters 5 and 6 as myCAF-like clusters, and cluster 7 as an ambiguous myCAF/iCAF/apCAF -like cluster. Cluster-specific genes were extracted from seven clusters, and the GO enrichment analysis revealed functional differences between each CAF cluster (Fig. S4d–e). In addition, IHC was performed using the extracted cluster markers, and their presence in both clinical PDAC and Sr-CDX was confirmed (Fig. 4g–h).

## Discussion

We evaluated the potential of AD-MSCs in providing heterogeneous CAF populations, as well as its contribution in generating a stroma-rich TME, with the CAF differentiation into multiple subtypes. Hitherto, PSCs and BM-MSCs have been considered as CAF precursors, and have been of interest. It has been proved that PSCs interact with cancer cells as well as with other cellular elements in the stroma, including immune cells, endothelial cells and neuronal cells, to set up a growth permissive microenvironment for PDACs^29^. In addition, recent studies have shown that PSCs possess the ability to differentiate into CAF subtypes, myCAF and iCAF^11^. BM-MSCs have shown the capability to adhere to the tumor site and promote tumor growth^18,20^, and be involved in the acquisition of chemotherapy resistance^21^. On the other hand, AD-MSCs have also been reported to differentiate into CAFs and be involved in tumor growth and desmoplastic reactions; however, the number of reports is relatively small^15,16^. Although GEMMs, such as the KPC (LSL-KrasG12D/+; LSL-Trp53R172H/+; Pdx-1-Cre) mouse model^30^, are excellent; verifying the relationships between the origin and heterogeneity of CAFs in those mice is difficult as well as clinical pancreatic cancer. In this study, immortalized AD-MSCs were efficacious in inducing CAF differentiation and succeeded in recapitulating the clinical PDAC characteristics, such as promoting tumor growth, drug resistance, and stromal desmoplasia, more accurately than the conventional in vivo models such as CDX, PDX, and GEMM.

RNA-seq analysis demonstrated that various CAF markers, including myCAF and iCAF markers, were differentially upregulated in Sr-CDX CAF (Fig. 3b). GO enrichment analysis, however, highlighted only the GO terms related to the myCAF functions (Fig. 3e). This discrepancy may be explained by the higher expression levels of myCAF markers in Sr-CDX CAFs than iCAF or apCAF markers. Therefore, CAF heterogeneity was examined further in our Sr-CDX models at the single-cell level.

**Figure 5:**
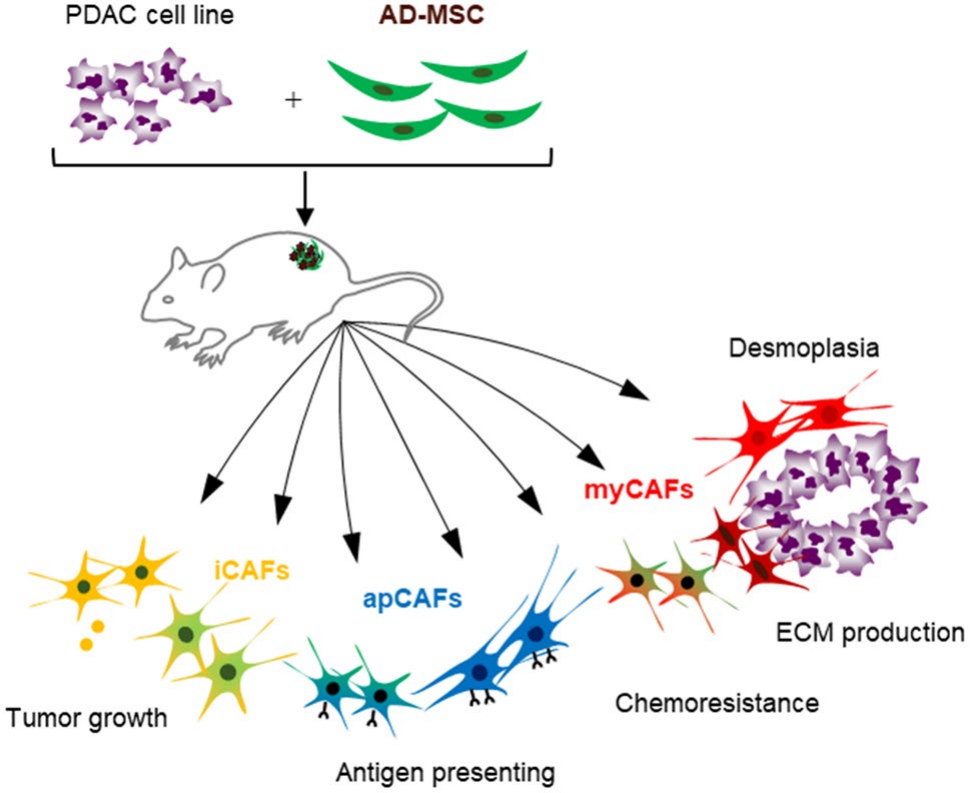
Schematic illustration of AD-MSC mediated stroma-rich cell line-derived xenograft model (Sr-CDX) of pancreatic cancer. In this study, AD-MSCs could differentiate into multiple subpopulations of CAFs, including myCAFs, iCAFs, apCAFs and other CAFs. The clinical features of pancreatic cancer, such as induction of vigorous tumor growth, stromal desmoplasia, CAF heterogeneity, and chemotherapy resistance, were well recapitulated in this Sr-CDX model.

Pioneering studies have already suggested the existence of multiple subpopulations of CAFs with distinct transcriptional profiles^10,31–33^. The presence of three representative subpopulations of CAFs was also reconfirmed in the CAFs of Sr-CDX models, as demonstrated by the presence of roughly two groups related to myCAF (clusters 5–7) and iCAF (clusters 1–4); additionally, the apCAF population overlapped mainly with the iCAF population (Fig. 4d–e). The presence of a fourth subtype of CAF, a purported mixture of myCAFs and iCAFs, has been proposed by Neuzillet et al. and named subtype C^9^; this subtype was visible both in the scRNA-seq analysis (cluster 7) and in IHC analysis (Fig. 2g,h, and 4d–h). In addition to the roles of myCAFs and iCAFs, the GO analysis indicated that the cluster 7 CAFs may be involved in "immune responses" that are associated with antigen presentation. Among the iCAF clusters containing four subclasses, CAFs belonging to the cluster 1 are enriched with apoptosis-related GO terms and may play a role in preventing cancer cell apoptosis and assisting cancer cell growth. It may have been related to tumor growth and chemotherapy resistance in the Sr-CDX mice (Fig. 1a–d and 5). CAFs in clusters 4 and 5 with enriched mitochondria-related GO terms may demonstrate activated metabolism and resistance to hypoxia and chemotherapy. CAFs in clusters 5–7 enriched with ECM-related GO terms may promote the production of abundant ECM, resulting in abundant stroma of the tumor in Sr-CDX and desmoplasia in clinical PDAC (Fig. 2a and 5). Notably, our Sr-CDX model contains a heterogeneous subpopulation of CAFs despite using only AD-MSCs as the source of CAFs, providing an excellent system for investigating CAFs. Considering that AD-MSCs alone did not form tumors in vivo, AD-MSCs could have differentiated into CAFs by interacting with Capan-1 cells. As we have shown in our in vitro co-culture experiments^27^, CAFs could be differentiated by contact or non-contact interaction with Capan-1. In that case, TGFβ and other factors secreted by cancer cells would be expected to be one of the factors that cause AD-MSCs to differentiate into CAFs^11,34^. In addition, cancer cells are expected to be more diverse in vivo TME compared to *in vitro*^35^. The heterogeneous and diverse CAFs generated from AD-MSCs in this study are considered to be differentiated by various signals from diverse cancer cells or by signals with concentration gradients in the tumor microenvironment.

These results could be attributed to the use of immortalized AD-MSCs. In fact, human telomerase reverse transcriptase (hTERT) immortalized fibroblasts have been used to retain important phenotypic traits in breast cancer research^36^, and also are reported to be useful to investigate the heterogeneous functions and phenotypes of CAFs^37^. The use of immortalized cells may allow human CAFs to maintain its heterogeneity and important functions in the tumor.

In conclusion, our Sr-CDX model, using the AD-MSCs, accurately reproduced the pathological features, chemotherapy resistance, and CAF heterogeneity observed in the clinical PDAC, and confirmed that the AD-MSCs can differentiate into multiple subpopulations of CAFs (Fig. 5). This novel Sr-CDX model has the potential to elucidate the mechanism of CAF differentiation and explain the contribution of CAFs in the progression of pancreatic cancer.

## Methods

### Cells and culture conditions

The immortalized human AD-MSC cell line ASC52telo (ATCC SCRC-4000) and the human pancreatic cancer cell lines Capan-1 (ATCC HTB-79) and MIAPaCa-2 (CRL-1420) were utilized as per the previous reports^22,27^. The isolation and culture conditions for cell lines and CAFs from the primary tumors were described previously^27^. Briefly, AD-MSCs were labeled with GFP, whereas Capan-1 cells were labeled with RFP via lentiviral transduction. HEK293T cells were seeded to a 6 well plate and co-transfected with pLKO.1-puro eGFP (Sigma-Aldrich, St. Louis, MO, USA), psPAX2 and pMD2.G at a ratio of 2:1.5:1.2 μg/well. Twenty-four hours later the viral supernatant was collected and filtrated with 0.45 μm membranes. The AD-MSCs were infected with the viral supernatant in the presence of Polybrene (10 μg/ml) for 24 h. After 72 h, puromycin was added to the medium to purify GFP-positive cells. To establish the clinical CAFs isolated from PDAC patients, PDAC tumor sections were minced and two or three 2-mm tumor pieces were plated onto a gelatin-treated 3.5-cm dish in HFDM-1 medium (Cell Science & Technology Institute, Sendai, Japan) supplemented with 1% streptomycin–penicillin and 5% FBS. The dishes were then incubated under 5% CO_2_, 20% O_2,_ at 37 °C. After a couple days of incubation, fibroblasts grew around the tumor fragments, and then the tumor fragments were removed. When an enough number of cells were observed, they were passaged or stored. Under these culture conditions, CAFs selectively grown, while the remaining PDAC cells were depleted following a few passages.

### Patient sample collection

Human PDAC tissue was obtained with patient consent. This study was approved by the Research Ethics Board of the University of Tsukuba and the Ethics Committee of the National Institute of Advanced Industrial Science and Technology (AIST) in accordance with the Declaration of Helsinki. Approval was obtained from the Tsukuba Clinical Research & Development Organization (T-CReDO protocol number: H25-119, R01-193) and the Ethics Committee of AIST (hi2019-0317-B). A total of six resected pancreatic cancer specimens were used in this study. For the application of these clinical samples to research purposes, written informed consent was obtained from all patients.

### Mouse model and in vivo experiments

Eight-week-old female nude mice (Balb/c nu/nu) were purchased from Japan CLEA Inc. (CLEA Japan, Tokyo, Japan) for use in this study. Mice were bred and housed under specific pathogen-free conditions at the Animal Center of AIST and University of Tsukuba and were randomized into two groups: Sr-CDX group and conventional CDX group (control group, Capan-1 only). In the CDX model, 1 × 10^6^ AD-MSCs and 1 × 10^6^ Capan-1 cells were subcutaneously transplanted into the mice, and the sizes of the generated tumors were monitored. After testing various ratios, the mixing ratio of AD-MSC and Capan-1 was determined to be 1:1. The tumor volume and weight of Sr-CDX group (n = 3) were compared with those of conventional CDX group (n = 3). Tumor volume was measured three times a week after transplantation, and tumor weight was measured at the time of sacrifice. Tumor volume (*V*) was then calculated using the following formula: *V* = 1/2 (*L* × *W* × *W*), where L is the largest tumor diameter and W is the smallest tumor diameter. To evaluate the tumor response to chemotherapy treatment, 20 mg/kg gemcitabine hydrochloride (#4548995063564, Wako) in 100 µL PBS was intraperitoneally injected three times (days 17, 19, and 21) following transplantation in the Sr-CDX group (n = 3) and conventional CDX group (n = 3). The mice were sacrificed on day 23, and all subcutaneous tumors were excised. All invasive procedures were performed under inhalation anesthesia with isoflurane. Euthanasia was performed by cervical dislocation after isoflurane inhalation anesthesia. All animal experiments and procedures were approved by the Institutional Animal Care and Use Committee of the respective institutes of AIST (A2020-310) and the Ethics Committee of University of Tsukuba (19-028), and were carried out in accordance with the approved guidelines. The study was carried out in compliance with the Animal research: Reporting in vivo experiments (ARRIVE) guidelines^38^.

### Immunohistochemical/immunofluorescent tissue staining

All staining was performed on 2-µm-thick mouse tissue sections and 3-µm-thick human tissue sections. Hematoxylin and eosin (H&E) staining and MT staining were performed according to the standard protocols. The primary antibodies used for IHC/immunofluorescent (IF) staining were as follows: IL-6 (1:400, ab9324; Abcam), GFP (1:500, #598; Medical & Biological Laboratories), αSMA (1:400, ab5694; Abcam), NCAPG (1:200, 24563-1-AP; Proteintech), PTX3 (1:50, ab90806; Abcam), COMP (1:200, ab74524; Abcam), NCAM1 (1:100, ab133345; Abcam).

### RNA sequencing (RNA-seq)

Total RNA was isolated using TRI Reagent (Molecular Research Center, Inc., Cincinnati, OH, USA), according to the manufacturer’s instructions. The library preparation and sequencing were conducted at Macrogen Japan using a Truseq library prep kit and NovaSeq 6000 (Illumina, San Diego, CA, USA) to produce 150-bp paired-end reads. The acquired data from two independent devices for each condition were mapped and quantified using STAR (2.7.1a) and RSEM (1.3.1). hg38 was used as the reference genome, and Ensemble GRCh38 was used for the gene annotation^39,40^. Subsequently, differentially expressed genes were analyzed by iDEP.91^41^. To investigate the function of the differentially expressed genes, GO enrichment analysis was performed using the database for annotation, visualization, and integrated discovery (DAVID)^42,43^. The raw sequences in FASTQ format were deposited at DNA Data Bank of Japan (DDBJ, accession number DRA010287).

### Sample preparation, staining, and sorting for single-cell RNA-seq

Tumor specimens from cell-derived xenograft mice (n = 3) were exercised on day14 and minced and enzymatically digested in 10% FBS/DMEM supplemented with Collagenase D (2.5 mg/mL, #11088866001, Roche, Basel, Switzerland) and DNase I (0.2 mg/mL, #11284932001, Roche) for 45 min at 37 °C. Following digestion, the cells were sorted using FACS-Aria III (BD Bioscience, Franklin Lakes, NJ, USA) according to the manufacturer’s instructions and collected in 10% FBS/DMEM. Following the exclusion of debris, DAPI (4′,6-diamidino-2-phenylindole)-negative and GFP-positive cells were collected. Finally, up to 12,000 cells per lane were loaded on 10X Chromium microfluidic chips for single-cell data processing.

### Single-cell data processing and analysis

The single-cell RNA-seq library was constructed using Chromium Controller and Chromium Single Cell 3′ Reagent Kits v3.1 (#CG000204, 10× Genomics, Pleasanton, CA, USA) according to the manufacturer’s protocol. The library was sequenced using NovaSeq6000 (Illumina) in accordance with the manufacturer’s instructions to obtain 100-bp paired-end reads. After sequencing, FASTQ files were generated using Cell Ranger ver. 2.1.0 mkfastq (10× Genomics). Dimensionality reduction with the t-distributed stochastic neighbor embedding (t-SNE) algorithm for visualization and k-means clustering was performed using Cell Ranger. The raw sequences in FASTQ format are available on DDBJ (accession number DRA010288).

### Statistical analysis

Data are represented as the mean ± standard deviation (SD). The differences between each group were compared using unpaired two-tailed Student's t-tests or one-way analysis of variance (ANOVA) with Tukey's multiple comparison test. All data were evaluated using ANOVA in the statistical analysis software package SPSS version 25.0 (IBM SPSS Statistics). A value of *P* < 0.05 was considered statistically significant. The error bars in the figures represent the SD.

## Supplementary Information

Supplementary Information 1.

Supplementary Information 2.

Supplementary Information 3.

Supplementary Information 4.

Supplementary Information 5.

Supplementary Information 6.

Supplementary Information 7.

Supplementary Information 8.

## Data Availability

RNA-seq data are available in the DDBJ Sequence Read Archive under accession number DRA010287 and DRA010288. The raw data are available from the corresponding author upon reasonable request.
