# Supplementary figures and images for: Adipose-derived mesenchymal stem cells differentiate into heterogeneous cancer-associated fibroblasts in a stroma-rich xenograft model
Source: Sci Rep. 2021 Feb 25;11:4690. doi: 10.1038/s41598-021-84058-3 (PMC7907195; doi:10.1038/s41598-021-84058-3)

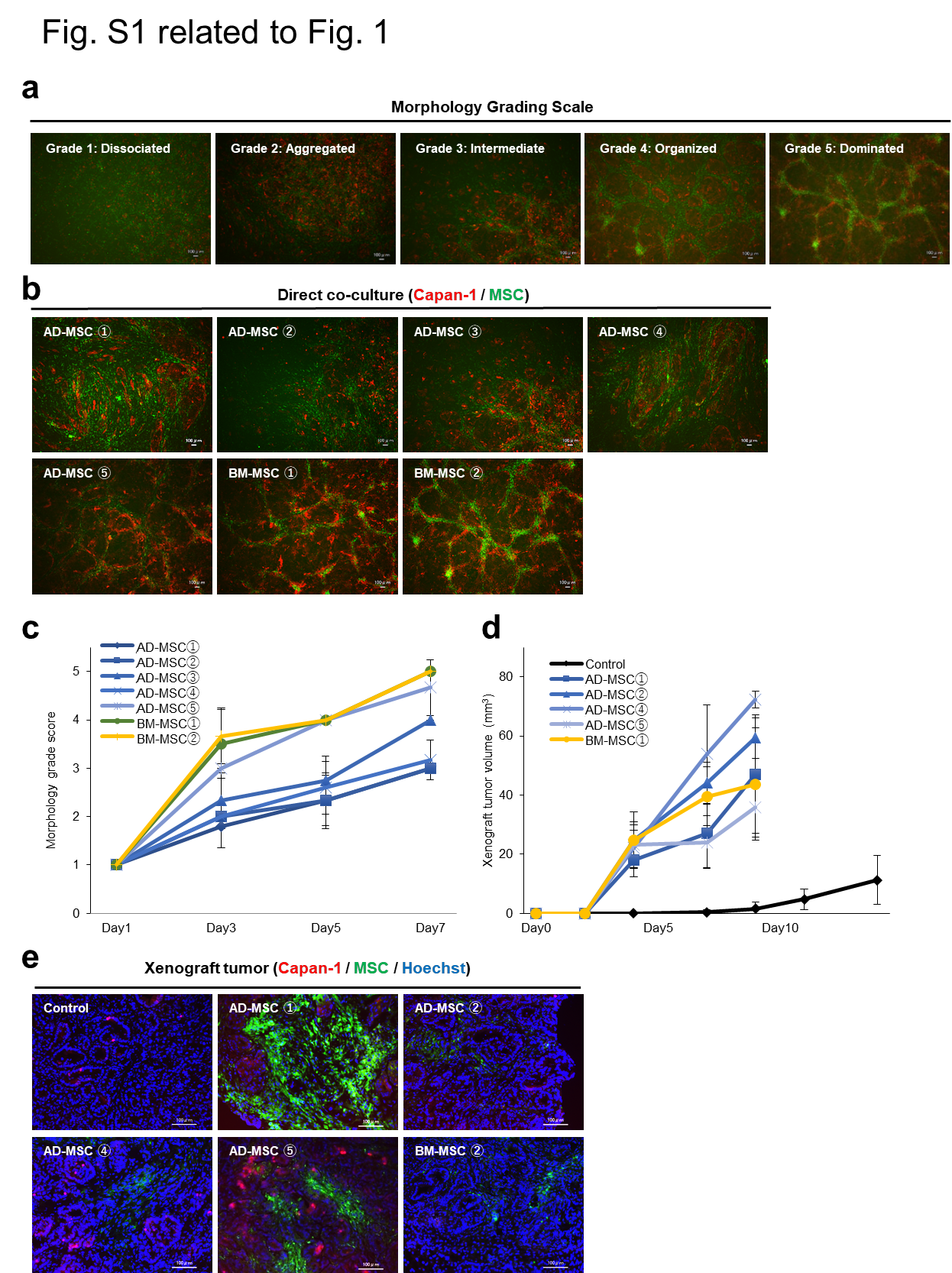

Supplement: Supplementary file 1 — Supplementary Information 1. [file 41598_2021_84058_MOESM1_ESM.tif]

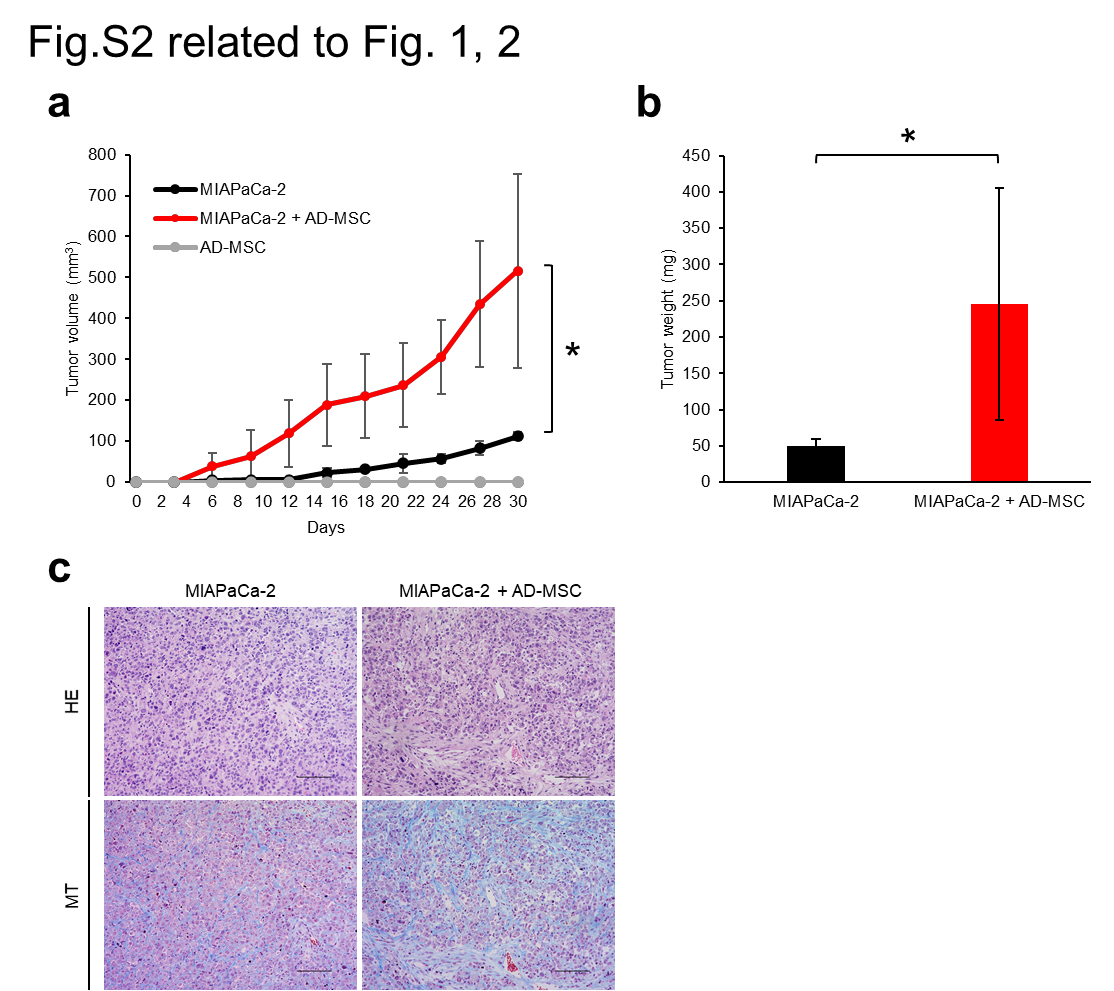

Supplement: Supplementary file 2 — Supplementary Information 2. [file 41598_2021_84058_MOESM2_ESM.tif]

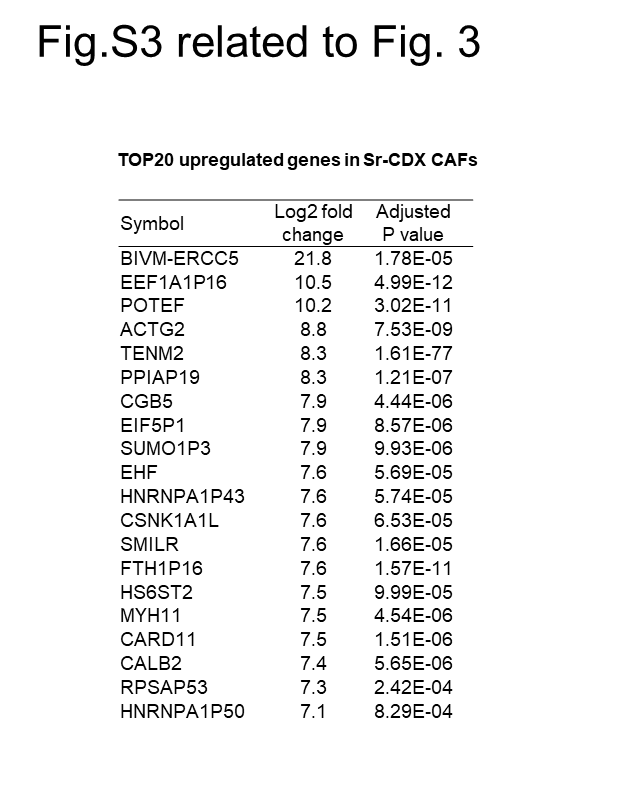

Supplement: Supplementary file 3 — Supplementary Information 3. [file 41598_2021_84058_MOESM3_ESM.tif]

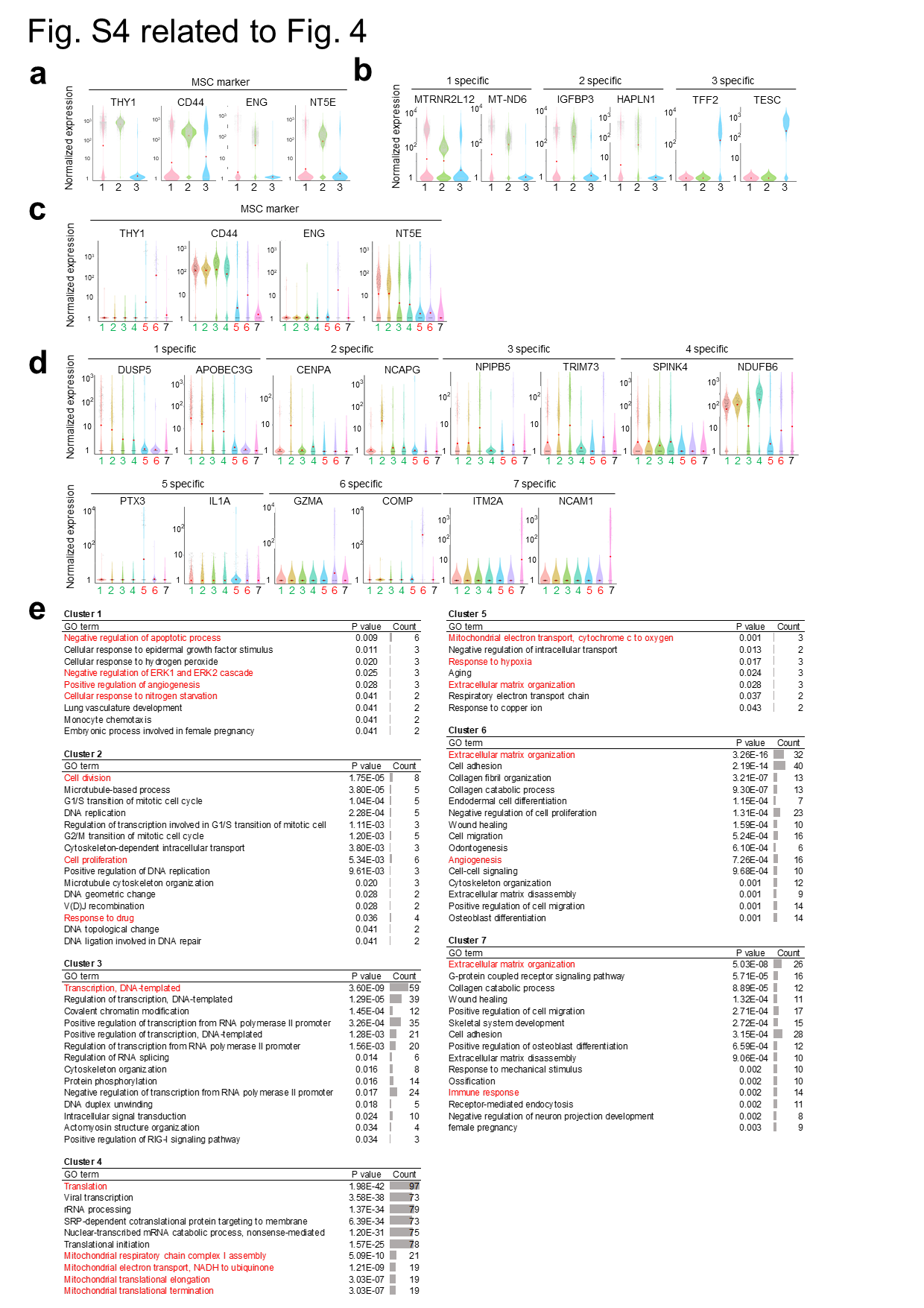

Supplement: Supplementary file 4 — Supplementary Information 4. [file 41598_2021_84058_MOESM4_ESM.tif]
