# Supplementary material for: Adipose-derived mesenchymal stem cells differentiate into heterogeneous cancer-associated fibroblasts in a stroma-rich xenograft model
Source: Sci Rep. 2021 Feb 25;11:4690. doi: 10.1038/s41598-021-84058-3 (PMC7907195; doi:10.1038/s41598-021-84058-3)
Supplement: Supplementary file 5 — Supplementary Information 5. [file 41598_2021_84058_MOESM5_ESM.docx]

**Supplementary information**

**Title:** **Adipose-derived mesenchymal stem cells differentiate into heterogeneous cancer-associated fibroblasts in a stroma-rich xenograft model**

**Authors’ names:** Yoshihiro Miyazaki^1,2^, Tatsuya Oda^1^, Yuki Inagaki^1,2^, Hiroko Kushige^2^, Yutaka Saito^3,4,5^, Nobuhito Mori^2^, Yuzo Takayama^2^, Yutaro Kumagai^2,6^, Toutai Mitsuyama^4^, Yasuyuki S. Kida^2,6*^

**Authors’ affiliations:**

^1^Department of Gastrointestinal and Hepato-Biliary-Pancreatic Surgery, Faculty of Medicine, University of Tsukuba, 1-1-1 Tennodai, Tsukuba, Ibaraki 305-8575, Japan.

^2^Cellular and Molecular Biotechnology Research Institute, National Institute of Advanced Industrial Science and Technology (AIST), Central 5-41, Higashi 1-1-1, Tsukuba 305-8565 Ibaraki, Japan.

^3^Artificial Intelligence Research Center, National Institute of Advanced Industrial Science and Technology (AIST), 2-4-7 Aomi, Koto-ku, Tokyo 135-0064, Japan.

^4^AIST-Waseda University Computational Bio Big-Data Open Innovation Laboratory (CBBD-OIL), 3-4-1 Okubo, Shinjuku-ku, Tokyo 169-8555, Japan.

^5^Graduate School of Frontier Sciences, University of Tokyo, 5-1-5 Kashiwanoha, Kashiwa, Chiba 277-8561, Japan.

^6^Advanced Photonics and Biosensing Open Innovation Laboratory, National Institute of Advanced Industrial Science and Technology (AIST), Central 5-41, Higashi 1-1-1, Tsukuba 305-8565 Ibaraki, Japan.

* **Corresponding author**

Yasuyuki S. Kida

Cellular and Molecular Biotechnology Research Institute, National Institute of Advanced Industrial Science and Technology (AIST), Central 5-41, Higashi 1-1-1, Tsukuba 305-8565 Ibaraki, Japan.

Tel: +81-29-861-3000

Fax: +81-29-861-3000

Email: y-kida@aist.go.jp

**Supplementary figure legend**

**Fig. S1. Various MSCs were differentiated into CAFs by *in vitro* co-culture and *in vivo* co-transplantation, and their similarity to CAFs was examined. Related to Fig. 1.**

**(a-c)** Human PDAC cell line Capan-1 and various types of MSC were directly co-cultured *in vitro* for seven days, and their morphological alterations were graded according to “Morphology Grading Scale”. High grading score means the resemblance more closely to clinical cancer-stromal image.

**(a)** Standard images of “Morphology Grading Scale”. Criteria of each grade were as follows: Grade 1: Dissociated, MSCs and cancer cells show a dissociated pattern; Grade 2: Aggregated, Each cell tends to aggregate respectively but not structurally; Grade 3: Intermediate, Intermediate between Grade 2 and Grade 4; Grade 4: Organized, MSCs present netlike appearance and cancer cells form island-like structure; Grade 5: Dominated, Cancer cells are expansive growth dominantly to MSCs. Scale bars, 100 µm.

**(b)** Representative IF image of co-cultures at day7. Each plot shows for Capan-1 (red) and MSCs (green). Scale bars, 100 µm.

**(c)** Morphology grading scores of day1, day3, day5, and day7. Grading scale was evaluated at least three hot spots and averaged every two days. Every MSC had the potential to get to Grade3 or more.

**(d)** Xenograft tumor volume after co-transplantation of Capan-1 with various MSCs. Only Capan-1 was transplanted in the control group.

**(e)** Representative IF image of xenograft tumors. Each plot shows for Capan-1 (red), MSCs (green), and Hoechst 33342 (blue). Scale bars, 100 µm.

**Fig. S2. Co-transplantation of the human PDAC cell line MIAPaCa-2 with AD-MSCs promoted tumor growth and stromal enlargement but did not result in the formation of glandular ductal structures. Related to Fig. 1, 2.**

**(a)** The tumor growth curve and **(b)** excised tumor weights at day 30. Results show the mean ± SD (n = 4). *, *P* < 0.05, one-way analysis of variance (ANOVA) with Tukey's method.

**(c)** Representative images of H&E staining and Masson’s trichrome staining in the MIAPaCa-2 CDX model and the MIAPaCa-2 and AD-MSC CDX model. Scale bars, 100 µm.

**Fig. S3. RNA-seq analysis revealed CAF-like differentiation of AD-MSCs after injection into Sr-CDX mice. Related to Fig. 3.**

Top 20 upregulated genes in Sr-CDX CAFs compared to those in the original AD-MSCs.

**Fig. S4. scRNA-seq revealed the heterogeneity of Sr-CDX CAFs. Related to Fig. 4.**

**(a)** Mono-cultured original AD-MSCs; Violin plot shows expression of MSC marker genes in each cluster after clustering of AD-MSCs. Red plots indicate median expression level.

**(b)** Mono-cultured original AD-MSCs; Violin plot shows the representative gene-expression pattern of each cluster. Red plots indicate median expression level.

**(c)** Sr-CDX CAFs; Violin plot shows expression of MSC marker genes in each cluster after clustering of Sr-CDX CAFs. Red plots indicate median expression level.

**(d)** Sr-CDX CAFs; Violin plot shows representative gene expression of each cluster after clustering of Sr-CDX CAFs. Red plots indicate median expression level.

**(e)** GO terms (biological process) enriched in clusters 1–7. Representative GO terms that may explain the function of each cluster are highlighted in red. Annotated GO terms for each of the seven clusters were quite variable. In iCAF-like clusters (clusters 1–4), the representative GO terms annotated to each cluster were as follows: cluster 1, “negative regulation of apoptotic process” and “positive regulation of angiogenesis”; cluster 2, “cell division,” “cell proliferation,” and ” response to drug”; cluster 3, “transcription, DNA-templated” and “regulation of transcription, DNA-templated”; cluster 4, “translation” and “mitochondrial respiratory chain complex I assembly”. In myCAF-like clusters (clusters 5 and 6), the representative GO terms assigned to cluster 5 were “mitochondrial electron transport, cytochrome c to oxygen,” “response to hypoxia,” and “extracellular matrix organization,” and those assigned to cluster 6 were “extracellular matrix organization” and “cell adhesion.” Cluster 7 corresponded to overlapping clusters of iCAF, myCAF, and apCAF, and the annotated GO terms were “extracellular matrix organization” and “immune response.”

**Table S1. Differentially expressed genes heatmap in Sr-CDX CAF vs AD-MSC.**

**Table S2. k-means clustering of original AD-MSC, Sr-CDX CAF and two clinical CAFs.**

**Table S3. Results of GO enrichment analysis for each cluster.**
